# Pharmacotherapy for Behçet’s Disease and the Risk of Malignancy

**DOI:** 10.3389/fphar.2021.661150

**Published:** 2021-07-20

**Authors:** Mao-Xin Huang, Cai-Yun Wang, Jin-Yan Guo, Jian-Hao Li, Xiao-Hong Li, Jiang-An Zhang, Jian-Bin Yu

**Affiliations:** ^1^ Department of Dermatology, The First Affiliated Hospital of Zhengzhou University, Zhengzhou, China; ^2^ Department of Rheumatism, The First Affiliated Hospital of Zhengzhou University, Zhengzhou, China; ^3^ Department of Infection, The First Affiliated Hospital of Zhengzhou University, Zhengzhou, China

**Keywords:** cancer, pharmacologic interventions, cyclophosphamide, chronic disease management, Behçet’s disease

## Abstract

**Background:** Behçet’s disease (BD) is associated with an increased risk of cancer. Few reports have been published on the relationship between drug exposure and the risk of cancer in patients with BD. Herein, we explored the relationship between pharmacologic interventions for BD and the risk of cancer.

**Methods:** we carried out a retrospective nested case-control study in a cohort of BD patients from attending our institution. Among 1,148 patients, 22 cancer patients were individually 1:2 matched to 44 cancer-free controls. The following biochemical indicators were evaluated: routine blood analysis, liver and kidney function tests, inflammatory indexes, blood gas analysis, blood electrolyte and previous pharmacologic interventions to manage BD including systemic glucocorticoids, methotrexate, cyclosporine-A, azathioprine, cyclophosphamide (CYC), and thalidomide, which are considered the primary medicines used for the management of BD.

**Results:** Among the 22 BD patients with cancers, myelodysplastic syndrome (MDS) (22.72%) was the most common type. Furthermore, CYC administration was significantly higher in BD patients with cancer compared with the cancer-free matched control group. Further, we observed that complement 4 (C4) (odds ratio [OR] = 0.0001, 95% confidence interval [CI]: 0.001–0.065) and hemoglobin (Hb) (OR = 0.891, 95% CI: 0.795–0.998) levels were independent protective factors for predicting cancer risk in BD patients on multivariate analyses.

**Conclusion:** Our study revealed that CYC was associated with a high risk of cancer in BD patients. Furthermore, C4 and Hb are independent protective factors for oncogenesis in BD patients. These findings may provide references and suggestions for clinicians to select appropriate treatments and for the early recognition of high-risk patients to reduce cancer incidence in BD patients.

## Introduction

Behçet’s disease (BD), also known as Behçet’s syndrome, is a chronic, multisystem autoimmune vasculitis characterized by painful oral or genital ulcers, skin lesions, uveitis, and other manifestations (Emmi et al., 2019). BD is common in young adulthood, which diminishes the quality of life and even causes disability (Davatchi et al., 2017). Because of its obscure pathogenesis and recurrent symptoms, life-long intermittent medicinal treatments may often be required to control the disease (Akman-Demir et al., 2011). Systemic glucocorticoids (GCs) and immunosuppressive or cytotoxic agents including thalidomide, azathioprine (AZA), cyclophosphamide (CYC) and cyclosporine (CsA) are usually combined to treat refractory symptoms or multiple organ involvement (Lopalco et al., 2020). Although the symptoms of BD patients have effectively improved, the adverse reactions associated with long-term drug use given its chronic disease course are also of concern (Gurcan et al., 2020).

Researchers had explored the associations between BD and cancer risk through nationwide population-based studies in Korea, which revealed an increased risk of hematologic cancer due to BD (Han et al., 2017) and it coincided with the conclusion of a higher risk of site-specific cancers of BD in Taiwan (Wang et al., 2015). The underlying mechanism of increased cancer risk has been speculated to be associated with BD increased malignant cell transformation and carcinogenesis by the chronic inflammation of BD with a dysregulated immune response and microenvironment (Wang et al., 2019). Prolonged exposure to immunosuppressants including AZA, CYC, CsA et al. would affect cancer risk in BD patients. Studies that clarify the relationship between pharmacologic interventions and cancer risk in BD are scarce. Previous studies have indicated that treatment with immunosuppressants increased the risk of cancers (Solomon et al., 2014; Brand et al., 2014), while Crusz and Balkwill (2015) showed that immunomodulators could reduce cancer risk. Two recent Korean population-based studies reported that three-quarters of BD patients who used thiopurines developed hematological cancer and the use of thiopurines was associated with lower solid and overall cancer risk in BD patients (Han et al., 2017; Han et al., 2018). However, given the lack of an adequate number of BD patients with cancer and long-term follow-up duration of aforementioned studies, further investigation is needed.

To explore the relationship between long-term pharmacologic interventions and cancer risk in patients with BD, we conducted a retrospective nested case-control study to gather information about demographic characteristics, laboratory testing and drug treatment exposure of BD patients. Our results indicated that patients with BD taking CYC had an increased risk of cancer compared to those treated with other agents.

## Materials and Methods

### Study Design

This retrospective cohort study was conducted by retrieving the medical records of all individuals who were diagnosed with BD between May 1, 2011 and June 30, 2020 at the First Affiliated Hospital of Zhengzhou University. The inclusion criteria were as follows: 1) patients who fulfilled the BD diagnostic criteria indicated by the 2018 European League Against Rheumatism (Hatemi et al., 2018) and 2) patients who had received at least 1 month of pharmacological treatment for BD accompanied by more than a 1-year follow-up. Patients were excluded if 1) they had a diagnosis of prevalent cancers or premalignant lesions or 2) were aged less than 18 years at BD diagnosis. Finally, 1,148 patients were enrolled with BD as the primary diagnosis, and of these a total of 22 cases with malignancies were included in our study whose cancer diagnoses were verified by histological and cytometrical analyses. Participants were classified into a malignancy group and a control group. Our study was approved by the Ethical Committee of the First Affiliated Hospital of Zhengzhou University (Ethical Approval Number: 2021-KY-0033-002). All data linked to patient privacy were anonymous and patient’s written informed consent was waived because of the retrospective nature of the study.

### Clinical and Laboratory Examinations

The following demographical and clinical data were collected: age, sex, age at BD diagnosis, course of BD progression before malignancy diagnosis, type of malignancy, as well as chronic comorbidities including diabetes, hypertension and cardiovascular diseases (Sun et al., 2016). Furthermore, routine analysis of blood, liver and kidney function tests, inflammatory indexes, blood gas analysis, and blood electrolytes were assessed.

### Exposure to Medications

Information regarding BD patients’ drug exposure was collected from the date of BD diagnosis to the date of cancer diagnosis in the cancer group or the date of admission to the end of follow-up in the control group. For the six medications of interest, the application of drugs included GCs, MTX, CsA, AZA, CYC, and thalidomide.

### Propensity Score Matching

We applied propensity score matching to reduce selection bias when assessing the drug exposure on cancer risk. According to the propensity scores, a 1-to-2 match between the cancer participants and the cancer-free participants was conducted. The propensity scores were calculated by considering the following covariates of age, sex, duration of BD and comorbidities such as diabetes, hypertension, and cardiovascular diseases. Eventually, 22 cancer patients were individually 1:2 matched to 44 cancer-free controls.

### Statistical Analysis

Continuous variables were expressed as mean ± standard deviation or median. Categorical variables were presented as number or percentage and the comparisons between the cancer group and the control group were tested by an independent-sample *t*-test, Mann–Whitney *U*-test, Pearson Chi-square test, Chi-square with Yates’ correction, or Fisher’s exact test. Univariate and multiple logistic regression analyses were performed to identify independent risk factors for cancer incidence. Routine blood tests, liver and kidney function tests, inflammatory indexes, blood gas analysis, blood electrolyte and drug exposure were considered independent variables. BD patients with cancer were used as dependent variables. The results are reported as adjusted odds ratios (OR) with 95% confidence intervals (CI). Based on the results of logistic regression analysis, a nomogram was constructed to predict cancer risk of BD. Furthermore, calibration curves and decision curve analysis (DCA) were performed to measure the reliability of the nomogram. *p*-values < 0.05 were considered statistically significant. SPSS for windows (Version 20.00; Armonk, NY United States) and R software (Version 3.6.1, https://www.r-project.org/) were used in the analyses.

## Results

### Study Population

**FIGURE 1:**
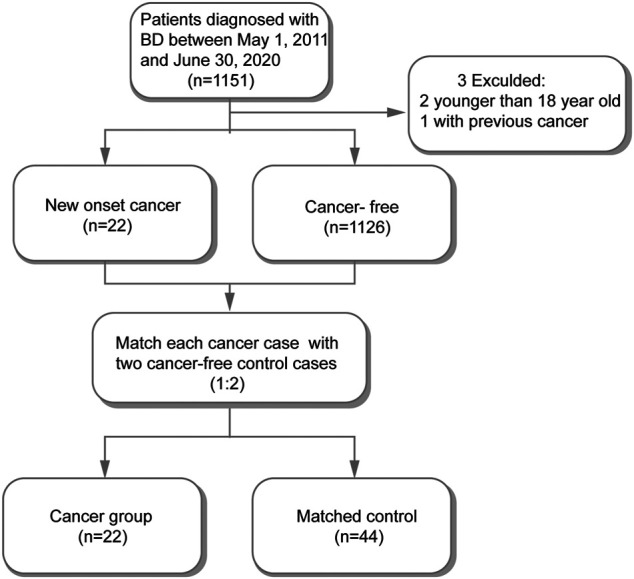
Flow chart of the study design.

Among the total of 1,151 hospitalized BD patients in our study, we excluded one patient who had prior to the BD diagnosis and two patients who were younger than 18 years old. The remaining 1148 BD patients were included in subsequent analyses among which 22 were cancer patients. We matched each cancer participants with two cancer-free participants (Figure 1). Therefore, 22 cancer patients and 44 matched cancer-free patients were enrolled in our study (Supplementary Table S1).

Before and after 1:2 propensity-score matching and matched patients’ baseline characteristics are compared in Table 1. Before matching, patients in the cancer group were older, had a longer duration of BD and were more likely to have a higher rate of cardiovascular disease. After matching, the distribution of baseline variables between the two groups was well balanced (Table 1).

**TABLE 1:** Characteristics of the study patients before and after propensity score.

Characteristic and comorbidity	Before matching			After matching		
	Cancer group (*n* = 22)	Control group (*n* = 1,126)	*p*-value	Cancer group (*n* = 22)	Control group (*n* = 44)	*p*-value
Age, median (year)	63.5	36	<0.001***	63.5	62.5	0.409
Female gender, *n* (%)	9, 40.91%	531, 47.16%	0.668	9, 40.91%	18, 40.91%	>0.999
Duration of BD, median (year)	8	3	<0.001***	8	8	0.551
Hypertension, *n* (%)	5, 22.73%	163, 14.48%	0.278	5, 22.73%	15, 34.09%	0.344
Diabetes, *n* (%)	6, 27.28%	155, 13.77%	0.071	6, 27.28%	15, 34.09%	0.575
Cardiovascular disease, *n* (%)	5, 22.73%	108, 9.59%	0.041*	5, 22.73%	12, 27.27%	0.691

Notes: *p < 0.05, **p < 0.01, ***p < 0.001.

Cardiovascular disease includes coronary artery diseases, atherosclerosis, hypertension and heart failure. Propensity score matching was performed for age, gender, duration of BD and chronic comorbidities (1:2 ratio).

### Comparison of Types of Malignancies and Laboratory Tests in the Cancer and Control Groups

**FIGURE 2:**
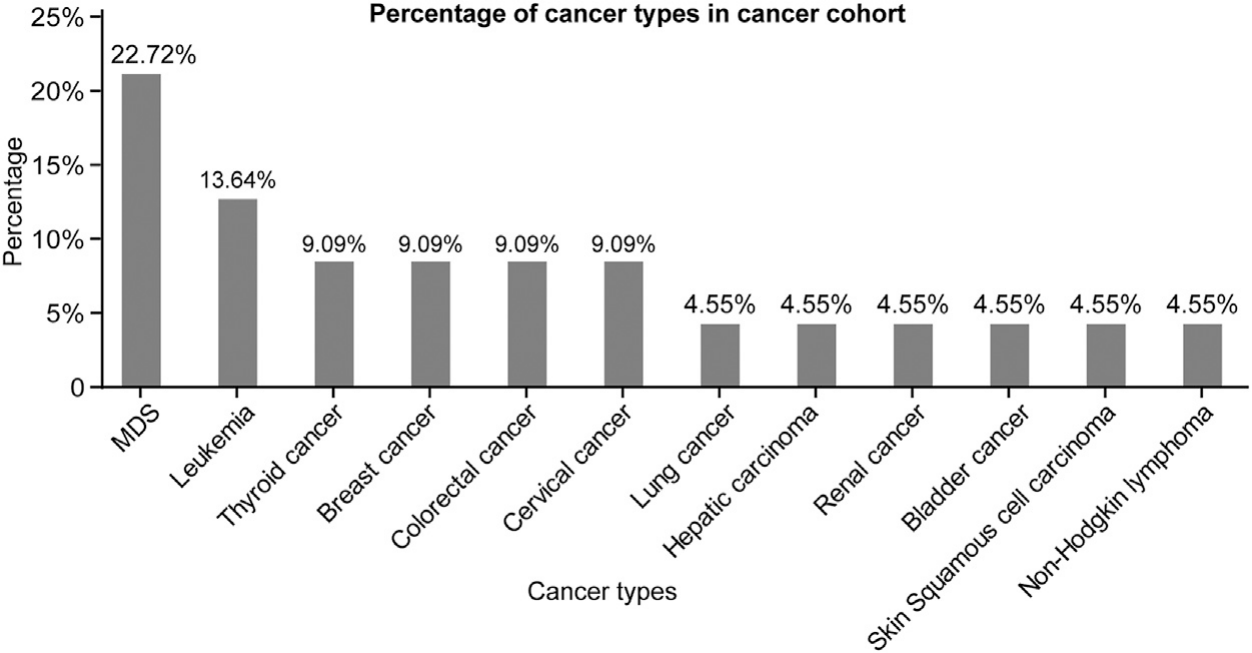
Percentage of cancer types in cancer cohort. **Abbreviations:** MDS, myelodysplastic syndrome.

The types of malignancies in our 22 BD patients are shown in Figure 2. Among the 22 cancer cases, nine patients developed hematological cancers (40.91%), and the most frequently observed diagnosis of cancer was myelodysplastic syndrome (MDS, 22.72%), followed by leukemia (13.64%) and non-Hodgkin lymphoma (4.55%). The remaining 13 patients presented solid cancer (59.09%) and included two thyroid cancers, two breast cancers, two colorectal cancers, two cervical cancers and one patient each with lung cancer, hepatic carcinoma, renal cancer, bladder cancer and skin squamous cell carcinoma (Figure 2). In addition, we analyzed the differences in laboratory tests between the cancer patients and the control patients. As shown in Supplementary Table S2, BD patients with malignancies had fewer red blood cells, platelets, lymphocytes, white blood cell counts, neutrophils, and lower hemoglobin (Hb), albumin, complement three and complement 4 (C4) than control patients. Meanwhile, they differed significantly in terms of the Na^+^, K^+^ and Ca^2+^ levels in arterial blood gas (*p* < 0.05) (Supplementary Table S2).

We provide insight into the understanding of variants in laboratory tests between cancer groups and control groups. Furthermore, to explore the possible factors influencing the risk of cancer in patients with BD, we performed univariate and multivariate logistic regression analyses (Table 2). In univariate logistic regression analyses, 12 independent variables were found to be significantly associated with cancer risk, and therefore were entered into the multivariate logistic regression analyses. Consequently, Hb (OR = 0.891, 95% CI: 0.795–0.998) and C4 (OR = 0.0001,95% CI: 0.001–0.065) levels were identified as independent protective factors for cancer risk in BD patients. These indexes may help clinicians to early recognize high-risk patients and reduce the cancer risk of BD patients.

**TABLE 2:** Univariate and multivariate logistic regression analyses.

Factors	Univariate analysis		Multivariate analysis	
	OR (95% CI)	*p*	OR (95% CI)	*p*
RBC	0.196 (0.077–0.404)	<0.001***	4.209 (0.229–77.399)	0.333
Hb	0.934 (0.898–0.961)	<0.001***	0.891 (0.795–0.998)	0.046*
WBC	0.829 (0.689–0.969)	0.029*	1.351 (0.291–6.278)	0.701
Lymph^#^	0.342 (0.135–0.699)	0.01*	0.649 (0.063–6.7)	0.717
Neut^#^	0.784 (0.616–0.969)	0.032*	0.616 (0.095–4.003)	0.611
Mono%	1.098 (1.017–1.22)	0.038*	0.916 (0.73–1.148)	0.445
ESR	1.031 (1.011–1.055)	0.005**	1.059 (0.962–1.166)	0.245
CRP	1.041 (1.017–1.072)	0.003**	1.053 (0.994–1.115)	0.079
C4	0.0001 (0–0.003)	0.003**	0.0001 (0.001–0.065)	0.023*
GLOB	1.122 (1.018–1.251)	0.027*	1.11 (0.936–1.317)	0.23
Na^+^	0.724 (0.592–0.85)	<0.001***	0.817 (0.618–1.08)	0.156
K^+^	0.251 (0.063–0.856)	0.036*	0.878 (0.109–7.047)	0.902

Notes: *p < 0.05, **p < 0.01, ***p < 0.001; Abbreviations: RBC, red blood cell count; Hb, hemoglobin; WBC, white blood cell count; Lymph^#^, Absolute value of lymphocyte; Neut^#^, Absolute value of neutrophils; Mono%, Percentage of monocytes; ESR, erythrocyte sedimentation rate; CRP, C-reactive protein; C4, complement 4; GLOB, Globulin; Na^+^, Na^+^ in arterial blood gas; K^+^, K^+^ in arterial blood gas.

### Exposure to Pharmaceutical Therapy Prior to the Development of Malignancy

**FIGURE 3:**
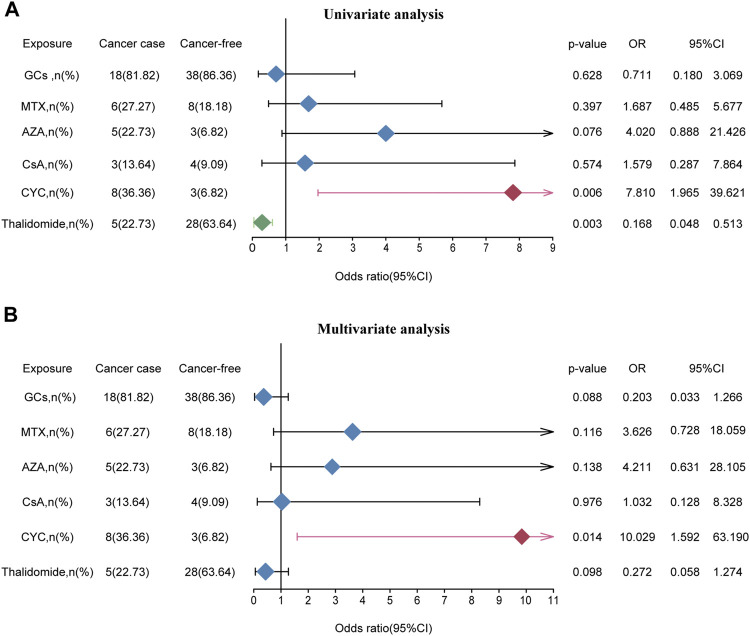
The effect of medication exposure on cancer risk in patients with BD. **(A)** Univariate analysis between medication exposure and cancer risk. **(B)** Multivariate analysis between medication exposure and cancer risk. **Abbreviations:** GCs, glucocorticoids; MTX, methotrexate; AZA, azathioprine; CsA, cyclosporine; CYC, cyclophosphamide; OR, odds ratio.

To further examine risk in drug exposure among BD patients with cancers, we perform the chi-square test (Supplementary Table S3) and logistic regression analyses (Figure 3). The univariate logistic regression analyses indicated that thalidomide treatment (OR = 0.168, 95% CI: 0.048–0.513) was as an independent protective factor for risk of cancer and CYC (OR = 7.810, 95% CI: 1.965–39.621) was associated with a high risk of cancer. The remaining agents including GCs (OR = 0.711, 95% CI: 0.180–3.069), MTX (OR = 1.687, 95% CI: 0.485–5.677), AZA (OR = 4.020, 95% CI: 0.888–21.426) and CsA (OR = 1.579, 95% CI: 1.965–39.621) did not significantly correlate with cancer risk. Moreover, thalidomide and CYC were entered into the multivariate logistic regression analyses, which revealed that CYC (OR = 10.029, 95% CI: 1.592–63.190) were independent risk factors for tumorigenesis (Figure 3).

### Establishment and Validation of a Nomogram for Predicting Cancer Risk of BD Patients

**FIGURE 4:**
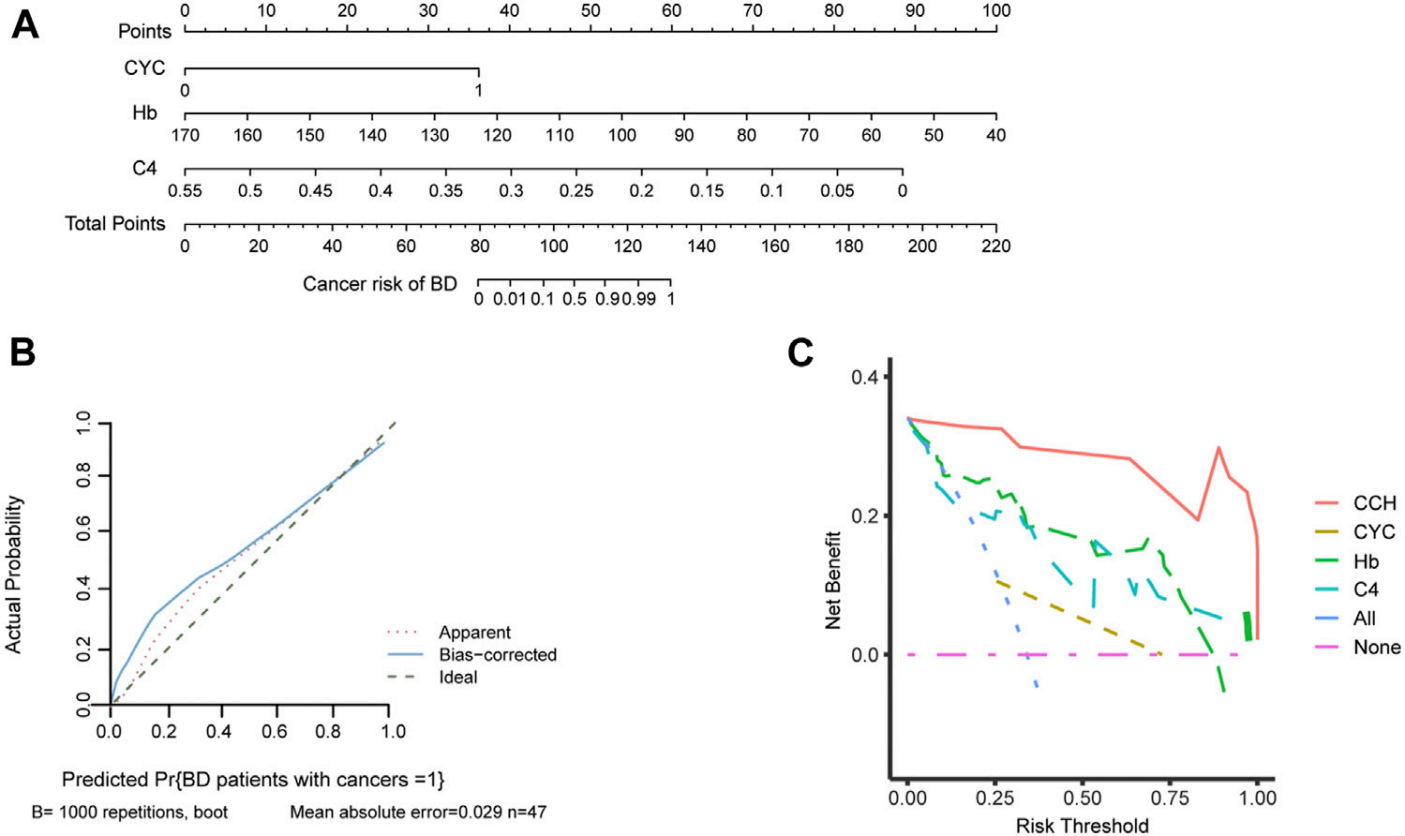
The establishment of nomogram to predict the cancer risk of BD patients. **(A)** Nomogram for predicting cancer risk of BD patients. Independent factors for cancer risk in patients with BD are located on each variable axis, and a line is drawn upward to determine corresponding scores. Three scores were added to obtain the total score, and a line is drawn downward to determine the cancer risk of BD patients. **(B)** Calibration curve of the nomogram. **(C)** A decision curve analysis (DCA) of the nomogram. Compared with single factor, our model may potentially yield more clinical net benefit in the risk threshold of 0.25–0.75. **Abbreviations:** CCH, cyclophosphamide-complement 4-hemoglobin; CYC, cyclophosphamide; C4, complement 4; Hb, hemoglobin.

To better evaluate the cancer risk of patients with BD, a nomogram was established based on independent factors for cancer risk in patients with BD (Figure 4A). We organized the C4, Hb and CYC into different points with each point corresponding to a score. For each patient, three scores were obtained for each variable, which were added to obtain the total score. The total score was used to predict the cancer risk of BD patients. Furthermore, the calibration curves exhibited sufficient agreement between the nomogram and actual probability of cancer risk of BD patients (Figure 4B). On DCA, our model indicated a higher threshold probability compared with the single factor to provide a superior estimation of decision outcomes (Figure 4C). With the risk threshold of 0.25–0.75, our model may potentially yield more clinical net benefit. In summary, our model could predict the cancer risk of BD patients.

## Discussion

Recently, Korean- and Taiwanese population-based studies have shown that BD patients have a high risk of cancer (Lin et al., 2014; Wang et al., 2015; Han et al., 2017; Han et al., 2018; Na et al., 2018). Several possible reasons might be closely related to high cancer risk, including immunosuppressant treatment administered for BD (Lin et al., 2014). To our knowledge, only few studies have mentioned the influence of medication use on the cancer risk. And among these studies the results were not completely convincing due to the limited number of BD patients for statistical power and incomplete medication information (Han et al., 2017; Han et al., 2018). Nonetheless, the relationship between drug exposure and the cancer risk of patients with BD has not yet been systematically analyzed.

Our study analyzed the relationship between medication exposure and the risk of malignancy among BD patients. In our cohort, the prevalence rate of malignancies was 1.92%, which was consistent with the Korean cohort and lower than a cohort of 651 BD patients in Beijing (Lin et al., 2014). And in previous studies, MDS was predominant among hematologic malignancies (Ahn et al., 2010). In addition, we found that patients with BD exposed to CYC were more prone to develop cancers. Consistent with our results, previous studies have demonstrated that CYC was a risk factor for several cancers such as non-melanoma skin cancers and bladder carcinomas (Faurschou et al., 2015). Overall, our findings may provide references and suggestions for clinicians for better selection of agents to reduce the cancer risk of BD patients.

Before biologics were available, patients with nervous system involvement and pulmonary artery involvement were prescribed CYC in the earlier years of the disease course (Hatemi et al., 2018). Despite its satisfactory therapeutic effects, CYC could cause toxicity. Short-term adverse events include gastrointestinal disturbances, hemorrhagic cystitis, alopecia bone marrow suppression, and infections (Brummaier et al., 2013), while the incidence of secondary malignancies and infertility are long-term and rare adverse events (Appel et al., 2009; Mok, 2016). Gurcan et al. (2020) recently found that BD patients treated with CYC were associated with a higher incidence of malignancy in a dose-dependent manner through a retrospective cohort study. In the present investigation, we also identified a higher risk of cancer in patients with BD who used CYC.

The mechanisms underlying this increased cancer risk in BD patients exposed to CYC are still poorly understood. It has been proposed that CYC might interfere with mitosis and induce DNA damage through the formation of DNA adducts (Hall and Tilby, 1992). Moreover, the hepatic metabolism of drugs including cytochrome P450 and genetic polymorphisms may result in adverse drug effects (Chen et al., 2019). Furthermore, patients with BD undergo multiple-drug therapy for prolonged periods and they may be more subjective to virally induced cancers (Sansone et al., 2014).

In our cohort, thalidomide was found not to be significantly associated with increase cancer risk on multivariate analysis, thus we think it can be a potential protective factor for tumorigenesis in BD patients. Thalidomide is effective for vasculitis and is broadly applied in BD treatment (Stirling, 1998). Besides its rapid improvement of oral and genital lesions, thalidomide can prolong the time of recurrence and reduce the severity of symptoms (Franks et al., 2004). Jin et al. (2013) proposed that thalidomide played a protective role in oral cancer among patients with lichen planus or chronic discoid lupus erythematosus. Some studies explored the mechanism of antitumor effects of thalidomide through its potent antiangiogenic, immunomodulatory, and anti-inflammatory properties. Further, thalidomide could suppress factors which lead to anti-angiogenic effects such as VEGF, bFGF, TNF-α and IFN-γ via the NF-kB signaling pathway (Sampaio et al., 1991; Keifer et al., 2001; Majumdar et al., 2002; Kucharczak et al., 2003). In addition, thalidomide activates T cells directly or by co-stimulating receptors and preventing the activity of tumor-derived immunosuppressive molecules, which increase NK cells’ cytotoxicity to kill tumor cells (Bartlett et al., 2004; Sleijfer et al., 2004). Finally, thalidomide inhibits prostaglandin synthesis via destabilizing mRNA of COX-2 to activate inflammatory responses (Jeng et al., 2003).

In this study, Hb and C4 levels were identified independent protective factor for cancer risk. Recent studies summarized that Hb level and the variation of C4 levels were correlated with BD activity (Zhang et al., 2019; Hou and Guan, 2020). Hou et al. showed that the high copy number of C4A can bring risks to BD pathogenesis by regulating the expression of C4A, downstream C4, thus enhancing the production of interleukin-6 (Hou et al., 2013). Zhang Z et al. inferred that the decline in Hb levels was related to BD activity, and Hb level could be used as an independent predictor of BD (Zhang et al., 2019). From a clinical standpoint, Hb and C4 were both of little value in predicting cancer risk. However, our model proposes that CYC exposure might increase the risk of cancer in BD patients especially in patients with low levels of Hb and C4.

Our study had a few limitations. Firstly, this was a single-center study and our findings need to be confirmed by future multi-center and prospective clinical studies. Furthermore, the drug mechanisms contributing to the cancer of BD remain largely unknown, which need further study.

## Conclusion

Our study revealed that CYC was associated with a higher risk of cancer in BD patients. Meanwhile, we found that C4 and Hb are independent protective factors for cancer risk in BD patients. Finally, we established a nomogram model suitable for clinical application.

## Data Availability

The original contributions presented in the study are included in the article/Supplementary Material, further inquiries can be directed to the corresponding authors.
