# Supplementary material for: Pharmacotherapy for Behçet’s Disease and the Risk of Malignancy
Source: Front Pharmacol. 2021 Jul 20;12:661150. doi: 10.3389/fphar.2021.661150 (PMC8329246; doi:10.3389/fphar.2021.661150)
Supplement: Supplementary file 1 [file DataSheet1.docx]

**Supplementary TableS1: Demographics of the cohort**

| Features | Cancer group  (n= 22) | Control group  (n= 44) |
| --- | --- | --- |
| Age |  |  |
| ＜59 years | 8 (36.36) | 18 (40.91) |
| ≥59 years | 14 (63.64) | 26 (59.09) |
| Mean age±SD | 57.05±16.49 | 60.11±12.84 |
| Gender |  |  |
| Male | 13 (59.09) | 26 (59.09) |
| Female | 9 (40.91) | 18 (40.91) |
| Duration of BD |  |  |
| ＜8 years | 10 (45.45) | 30 (68.19) |
| ≥8 years | 12 (54.55) | 14 (31.81) |
| Mean duration±SD | 8.32±4.77 | 6.98±5.09 |
| Hypertension |  |  |
| yes | 5 (22.73) | 15 (34.09) |
| no | 17 (77.27) | 29 (65.91) |
| Diabetes |  |  |
| yes | 6 (27.28) | 15 (34.09) |
| no | 16 (72.72) | 29 (65.91) |
| Cardiovascular disease |  |  |
| yes | 5 (22.73) | 12 (27.27) |
| no | 17 (77.27) | 32 (72.73) |

**Supplementary TableS2:** **Comparison of the laboratory tests between the two groups.**

| Laboratory features | Control group | Cancer group | P |
| --- | --- | --- | --- |
|  | Mean± SD | Mean± SD |  |
| RBC | 4.042 ± 0.117 | 2.768 ± 0.204 | <0.001*** |
| Hb | 123.2 ± 3.069 | 83.73 ± 5.758 | <0.001*** |
| PLT | 218.2 ± 13.26 | 168.6 ± 31.4 | 0.093 |
| WBC | 7.934 ± 0.4918 | 5.691 ± 0.9417 | 0.023* |
| Mono% | 7.972 ± 0.551 | 12.84 ± 2.455 | 0.013* |
| Neut# | 5.139 ± 0.372 | 3.622 ± 0.590 | 0.027* |
| Lymph# | 1.983 ± 0.177 | 1.185 ± 0.157 | 0.005** |
| ALB | 39.35 ± 0.778 | 36.53 ± 1.213 | 0.048* |
| GLOB | 25.21 ± 0.788 | 28.59 ± 1.237 | 0.021* |
| ESR | 23.39 ± 4.04 | 48.92 ± 6.798 | 0.001** |
| CRP | 10.75 ± 2.787 | 39.84 ± 8.31 | <0.001*** |
| C3 | 1.215 ± 0.041 | 1.009 ± 0.107 | 0.035* |
| C4 | 0.338 ± 0.015 | 0.217 ± 0.030 | <0.001*** |
| Na^+^ | 142.6 ± 0.458 | 137.7 ± 1.127 | <0.001*** |
| K^+^ | 4.252 ± 0.069 | 3.997 ± 0.088 | 0.030* |
| Ca^2+^ | 2.235 ± 0.025 | 2.15 ± 0.032 | 0.048* |

Notes: * p<0.05, ** p <0.01, ***p<0.001

Abbreviations: RBC, red blood cell count; Hb, hemoglobin; PLT, platelet count; WBC, white blood cell count; Mono%, Percentage of monocytes; Neut#, Absolute value of neutrophils; Lymph#, Absolute value of lymphocyte; ALB, albumin; GLOB, globulin; ESR, erythrocyte sedimentation rate; CRP, C- reactive protein; C3, complement 3; C4, complement 4; Na+, Na+ in arterial blood gas; K+, K+ in arterial blood gas; Ca2+, Ca2+ in arterial blood gas.

**Supplementary TableS3: Comparison of the pharmaceutical therapy between the two groups.**

| Exposure | Control group | Cancer group | p-value |
| --- | --- | --- | --- |
| GCs | 44 | 18 | 0.903 |
| MTX | 8 | 6 | 0.394 |
| AZA | 3 | 5 | 0.105 |
| CsA | 4 | 3 | >0.999 |
| CYC | 3 | 8 | 0.007** |
| Thalidomide | 28 | 5 | 0.004** |

Notes: * p<0.05, ** p <0.01, ***p<0.001

Abbreviations: GCs, glucocorticoids; MTX, methotrexate; AZA, Azathioprine; CsA, Cyclosporine A; CYC, cyclophosphamide.
